# Phase II trial of isotonic fluid resuscitation in Kenyan children with severe malnutrition and hypovolaemia

**DOI:** 10.1186/1471-2431-10-71

**Published:** 2010-10-06

**Authors:** Samuel O Akech, Japhet Karisa, Phellister Nakamya, Mwanamvua Boga, Kathryn Maitland

**Affiliations:** 1 KEMRI-Wellcome Trust Research Programme, Centre for Geographic Medicine Research-Coast, Kilifi, Kenya; 2 Department of Paediatrics, Faculty of Medicine, Imperial College, London, UK; 3 Wellcome Trust Centre for Clinical Tropical Medicine, Imperial College, London, UK

## Abstract

**Background:**

Children with severe malnutrition who develop shock have a high mortality. Contrary to contemporaneous paediatric practice, current guidelines recommend use of low dose hypotonic fluid resuscitation (half-strength Darrows/5% dextrose (HSD/5D). We evaluated the safety and efficacy of this guideline compared to resuscitation with a standard isotonic solution.

**Methods:**

A Phase II randomised controlled, safety and efficacy trial in Kenyan children aged over 6 months with severe malnutrition and shock including children with severe dehydration/shock and presumptive septic shock (non-diarrhoeal shock). Eligible children were randomised to HSD/5D or Ringer's Lactate (RL). A maximum of two boluses of 15 ml/kg of HSD/5D were given over two hours (as recommended by guidelines) while those randomised to RL received 10 ml/kg aliquots half hourly (maximum 40 ml/kg). Primary endpoint was resolution of shock at 8 and 24 hours. Secondary outcomes included resolution of acidosis, adverse events and mortality.

**Results:**

61 children were enrolled: 41 had shock and severe dehydrating diarrhoea, 20 had presumptive septic shock; 69% had decompensated shock. By 8 hours response to volume resuscitation was poor with shock persisting in most children:-HSD/5D 15/22 (68%) and RL14/25 (52%), p = 0.39. Oliguria was more prevalent at 8 hours in the HSD/5D group, 9/22 (41%), compared to RL-3/25 (12%), p = 0.02. Mortality was high, HSD/5D-15/26(58%) and RL 13/29(45%); p = 0.42. Most deaths occurred within 48 hours of admission. Neither pulmonary oedema nor cardiogenic failure was detected.

**Conclusions:**

Outcome was universally poor characterised by persistence of shock, oliguria and high case fatality. Isotonic fluid was associated with modest improvement in shock and survival when compared to HSD/5D but inconclusive due to the limitations of design and effectiveness of either resuscitation strategy. Although isotonic fluid resuscitation did not result in cardiogenic heart failure, as previously feared, we conclude that the modest volumes used and rate of infusion were insufficient to promptly correct shock. The adverse performance of the recommended fluid resuscitation guideline for severe malnutrition should prompt clinical investigation of isotonic fluids for resuscitation of compensated shock, defining rate and volumes required to inform future guidelines.

**Trial Registration:**

The trial is registered as ISCRTN: 61146418

## Background

Severe malnutrition is a common cause of admission to hospital in young children in Africa and outcome remains poor. The World Health Organization (WHO) 10-step treatment has improved case fatality, in some settings, to under 5%[1-3], however, in African hospitals implementation of the same guideline has achieved poorer results with numerous reports of unacceptably high case fatality rates[4,5]. At our hospital on the Kenyan coast, we have recently reported that mortality rates in children with severe malnutrition, treated in accordance with WHO guidelines, were 20%. Thirty percent of the fatalities occurred within 48 hours of admission and many had signs suggestive of hypovolaemic shock including children with severe dehydrating diarrhoea[4].

WHO malnutrition guidelines advocate strict avoidance of intravenous fluids and restrict the use of fluid resuscitation to children with advanced features of shock [1,6,7]. Fluid resuscitation is recommended only if *all *of the following parameters are present- a weak, fast pulse, cold peripheries, a capillary refilling time (CRT) of > 3 seconds plus signs of impaired consciousness (WHO malnutrition shock criteria)[6,7]. Ordinarily, these features would be considered by paediatric life-support providers as constituting a very advanced state of shock, when outcome is generally poor. For children fulfilling these criteria, preferential use of low-volume hypotonic fluids (0.45% sodium content) is recommended since it is commonly considered that malnourished children are at increased risk of developing congestive heart failure and sodium and water overload[8]. There is substantial debate over best treatment with scientific rationale advanced to justify this highly promoted WHO guideline, but what is clear is that the evidence base is weak and unsupported by the relevant physiological studies or clinical trials [8,9].

The criteria for shock and fluid management recommendations are a distinct departure from contemporary paediatric practice which advocates the recognition of an early phase of shock (compensated shock) and rapid correction with isotonic resuscitation fluids to restore circulatory volume. These have been implemented widely and prospectively evaluated. Children who were managed according to American College of Critical Care Medicine/Pediatric Advanced Life Support (ACCM-PALS) guideline[10,11] that received up to 60 mls/kg of isotonic fluid resuscitation over the first hour by community physicians or para-medics show a nine-fold reduction in mortality compared to cases who were not managed in accordance with these recommendations [12]. Importantly, these guidelines are widely practiced throughout the world, largely by non specialists but are still not standard practice in many African hospitals where they have yet to be evaluated.

In light of the high mortality of children with severe malnutrition and features of shock[4] we undertook a prospective evaluation of volume resuscitation in a pilot study (Fluid resuscitation In Malnutrition Trial: FIM). The FIM trial was conducted to examine the safety and efficacy of isotonic (0.9% sodium content) low volume fluid resuscitation and current WHO resuscitation protocol using low-volume hypotonic fluids. In this trial, we used isotonic fluids very cautiously, using similar volumes to those used WHO resuscitation protocol. In the absence of any safety data for this study population, the administration of appropriate volume resuscitation, as recommended internationally, was not incorporated in the design before the generation of the relevant haemodynamic data for which the risks and benefits could be assessed.

## Methods

### Participants

The study was conducted on the paediatric high dependency unit (HDU) Kilifi District Hospital (KDH), situated on the coast of Kenya. The amenities and expertise of the personnel enable full hemodynamic monitoring in critically ill children. However, no artificial ventilation facilities are available. Medically qualified members of the Kenya Medical Research Institute (KEMRI) team completed a standard admission questionnaire and examination at admission to hospital. Severe malnutrition (SM) was defined as any of: weight for height z-score <-3 or weight for height percentile 70%; or mid-upper arm circumference (MUAC) < 11.0 cm; or oedema involving at least both feet (kwashiorkor). MUAC was measured with a cloth (non-stretchable) measuring tape, weight with an electronic scale (Soehnle model 7300, CMS Instruments, UK), and length using a measuring board of standard design.

Children were eligible for inclusion in the study if they were aged over 6 months with severe malnutrition with evidence of shock. Prior to this trial we undertook a pilot evaluation of the WHO shock and fluid resuscitation guidelines in 8 children which resulted in universally fatal outcome. The chief reasons were considered to be that the WHO malnutrition shock criteria identified children with very advanced stages of shock and high risk of mortality. In addition was the poorly tolerated early initiation of feeding which is recommended by WHO guideline in children immediately after fluid resuscitation. For the Phase II feeding was withheld until children were stabilised and were able to tolerate nutritional supplementation. The stringent WHO shock criteria were amended to include children with one or more of the following: CRT > 2 seconds, lower limb temperature gradient, weak pulse volume, prolonged capillary refill > 2 seconds, deep 'acidotic' or 'Kussmaul' breathing, creatinine >80 μmol/L, or depressed conscious state (prostration (inability to sit up if aged >8 months) if present after correction of hypoglycaemia. Temperature gradient was defined as cooler extremities to warmer core and assessed by running the back of the palm of the hand up the lower limb. The radial pulse was used to assess pulse volume. Children were excluded if they had any of the following: severe anaemia (haemoglobin ≤5 g/dL); pulmonary oedema (defined as clinical evidence of presence of fine crepitations in both lung fields plus oxygen saturations < 90% in air); raised intra-cranial pressure or known congenital heart disease.

Individual informed written consent was obtained from parents/guardians before randomisation. When the situation was judged as an emergency, ethical approval permitted initial verbal assent followed by deferred informed consent once the child had been stabilised. Random allocation was assigned by the use of sealed cards and study interventions were not masked. Oxford University was the sponsor of the trial. The FIM trial is registered as ISCRTN: 61146418. Ethical approval for the study was obtained from the national ethics committee of KEMRI and OXTREC (Oxford Research Ethical Committee).

### Interventions

Two groups were considered. **Severe dehydration/shock **(shock and severe dehydrating diarrhoea defined as ≥6 watery stools per day) who were randomly assigned to receive either WHO fluid resuscitation regime (half-strength Darrow's in 5% dextrose (HSD/5D)) or Ringers Lactate (RL) and **Presumptive septic shock **(non diarrhoeal shock) randomised to one of three fluid resuscitation intervention arms: WHO fluid resuscitation regime, Ringers Lactate or 5% human albumin solution (HAS).

#### The WHO fluid resuscitation regime

initial bolus of 15 mls/kg of HSD/5D over one hour. Repeat bolus was given once (15 ml/kg of HSD/5D over 1 hour) if some improvement in features of shock noted. If no improvement was seen, they received 10 mls/kg whole blood transfusion over 3 hours.

#### Ringers Lactate or albumin resuscitation

initial bolus of 10 ml/kg over 30 minutes, repeated only twice over one hour (i.e. up to 30 ml/kg in total) if clinical reassessment demonstrated any of the following features of shock: CRT > 3 s, weak pulse volume, temperature gradient or hypotension (systolic blood pressure (SBP) <80 mmHg)).

Additional boluses (10 mls/kg over one hour) were only permitted if oliguria (< 0.5 mls/kg/hour) or hypotension (systolic pressure <80 mmHg) developed (20 mls/kg over one hour). Maximum bolus volumes given were 40 ml/kg. At each clinical review children were assessed for clinical resolution of shock and examined for signs of pulmonary oedema (if present further boluses withheld and treated with diuretics). No invasive monitoring, such as central venous pressure (CVP) measurement, was used. The children did not receive inotropes, vasopressors, or hydrocortisone. Other than the initial fluid boluses additional intravenous fluids boluses, intravenous rehydration for children with severe diarrhoea or maintenance fluids were not given (as per guideline recommendation). The only exception was if the child was intolerant to feeding when low volume maintenance was provided. Children were continuously and non-invasively monitored for heart and respiratory rate, oxygen saturation using a multi channel Siemens^® ^monitor and hourly for blood pressure and urine output then every 4 hours after 8 hours. At admission blood gases, plasma biochemistry, and haematology were assessed and reassessed at 8- and 24-hours post-admission. Blood and urine were cultured at admission on all children and lumbar puncture, where indicated. Adherence to protocol was validated by an internal but independent monitoring team. The trial was monitored three times during execution.

### Standard management of severe malnutrition

In all other respects children were treated according to WHO guidelines [13]. Hypoglycaemia (blood glucose <3 mmols/L) was treated with 5 mls/kg of 10% dextrose. Malnutrition oral rehydration solution (ReSoMal) [13] was given to children with significant diarrhoea (>6 loose stools/day) rather than intravenous rehydration irrespective of the level of clinical dehydration[14]. All children received intravenous ampicillin (50 mg/kg four times per day) and intramuscular gentamicin (7.5 mg/kg once daily) for at least 5 days. Ceftriaxone was used as second line antimicrobials or when directed by microbiological results. The macro and micro-nutritional aspects of management are covered in a previous publications[4]. Early nasogastric feeding, recommended by the guideline immediately after resuscitation was withheld, and children placed on maintenance intravenous dextrose fluids until children were stabilised, intestinal ileus excluded and tolerance of oral feeds established.

### Objectives

Primarily to establish whether hypovolaemic shock could be safely corrected by volume replacement Ringer's Lactate (an isotonic crystalloid), Human albumin solution (HAS) or HSD/5D (hypotonic crystalloid). The secondary objective was to assess the frequency of serious side effects, namely pulmonary oedema.

### Outcomes

Primary outcome measurement was the resolution of features of shock at 8 and 24 hours. Resolution of shock, defined as the absence of all of: severe tachycardia (heart rate >160 beats per minute), CRT > 2 s or oliguria (urine output<1 ml/kg/hr). Secondary outcomes included incidence of adverse events (such as fluid overload) and mortality.

### Sample size

The null hypothesis was that there is no difference in the safety profile or effect on physiological parameters of shock using Ringer's Lactate, HAS (isotonic crystalloids), or HSD/5D (hypotonic crystalloid) for fluid resuscitation. Our aim was to generate data to address the trial objectives balanced against the desire to minimize exposure of children to a therapeutic intervention for which there is no published data. We aimed to recruit 90 children: 45 to receive Ringer's Lactate, 45 to receive HSD/5D, and 20 to receive HAS. The numbers were chosen to provide sufficient information on haemodynamic response and adverse events to the two fluid management regimes to understand potential efficacy of each regime rather than compare the two strategies.

### Statistical methods

Dichotomous and categorical variables were created from continuous variables. Derived variables were created from clinical factors defined by Paediatric Advanced Life Support (PALS) guidelines as indicating a definitive need for urgent therapeutic intervention and for laboratory variables. We defined the following: severe tachycardia (>160 beats/minute); bradycardia (<60 beats/min); hypoxia (oxygen saturation <95% on air or unable to record by pulse oximeter); tachypnoea (respiratory rate>60 breaths/minute at any age); hypothermia (axillary temperature < 35°C); capillary refill ≥3 seconds; hypotension (systolic blood pressure <70 mmHg or unrecordable); acidosis (base deficit >8); elevated creatinine (>80 μmols/l); hypoglycaemia (<3.0 mmols/l); hyperglycaemia (>10 mmols/l); hypokalaemia (<3 mmols/l); hyperkalaemia (>5.5 mmols/l) or hyponatraemia (<125 mmols/l). Means and standard deviations were calculated for continuous variables using the student t-tests. Non-normally distributed data were compared using Sign-rank test and Kruskal-Wallis. Proportions were compared using chi-square and Fisher's exact tests as appropriate. We also used Kaplan Meier survival analysis to compared time to event (death). The area under curves (AUC) were calculated for serial measurements and their medians compared using Wilcoxon-Ranksum and Kruskal-Wallis tests. We employed AUC in order to compensate for confounding effect of early mortality, hence missing observations, leading to biases in the highest risk group and resulting imbalance within the survivors[15]. All analyses were by intention to treat.

## Results

**Figure 1:**
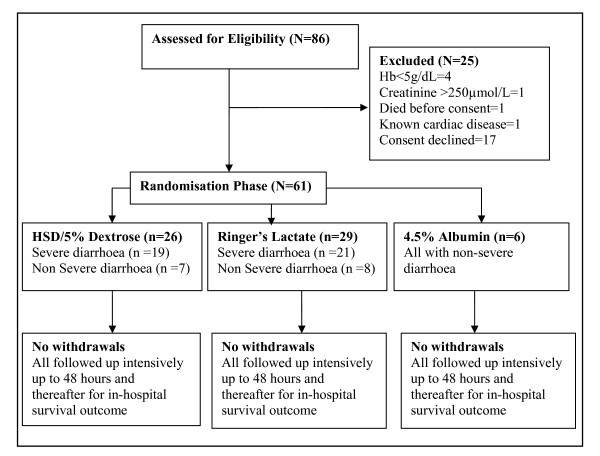
Trial flow

Eighty six severely malnourished children with shock were assessed for eligibility. Of these 25 were ineligible for recruitment; 61 were enrolled (Figure 1: trial flow). Forty children had hypovolaemic shock secondary to dehydrating diarrhoea and 21 had hypovolaemic shock, presumed to have been secondary to sepsis or a sepsis-like syndrome. None of the children excluded had cardiogenic shock or pulmonary oedema. Only six children with presumptive septic shock received 4.5% albumin. Baseline characteristics (and haemodynamic responses) of these 6 individuals were similar to the other participants in the sepsis group that were randomised to the other two investigational fluids but are not summarised due to small numbers. Recruitment commenced in November 2006 and the decision to discontinue recruitment was made after interim review of the safety data in May 2008.

### Numbers analysed

Sixty one children were recruited, 26 received HSD/5D, 29 received RL and 6 HAS. Forty had severe dehydration/shock diarrhoea (RL = 22, HSD/%D = 19) and 21 has presumptive septic shock (RL = 8, HSD/%D = 7, HAS = 6). By intention to treat, we report results of primary outcome comparisons between 26 children who received HSD and 29 who received RL. Owing to the small numbers who received HAS, we only report mortality and safety outcome for those treated with HAS.

### Baseline data

The median age of the trial participants was 15 months (interquartile range; 12, 23); 64% (35) had severe marasmus and 21% (13) had features of oedematous malnutrition (kwashiorkor). Overall, 75% (41) fulfilled the strict WHO definition of advanced shock. Baseline characteristics and disease severity indices were similar across the fluid intervention arms (Table 1). Children with shock and dehydration secondary to diarrhoea 32/40 (80%) had a higher frequency of WHO severe malnutrition shock definition than children with presumptive sepsis, 10/21 (48%) (p = 0.01). The diarrhoeal group were also more severely acidaemia (pH 7.22 ± 0.19 versus 7.34 ± 0.17; p = 0.03). The mean volume for the bolus infused in children randomised to HSD/5D was 30 ml/kg (standard deviation ± 10 ml) and 39 ml/kg (standard deviation ± 22 ml) in those receiving RL.

**Table 1:** Baseline characteristics for children in Phase II trial

		HSD/5D (n = 26)	RL (n = 29)	P
**Male, n (%)**		15(58)	17(59)	0.94
**Age, months (IQR)**		15(14)	16(6)	0.41
**Feature**	**N (%)**			
	MUAC, mean ± SD	10.4(1.4)	10.0(1.9)	0.43
	WHZ, mean ± SD	-3.4(1.3)	-3.9(1.0)	0.18
	Severe wasting	14(54)	21(72)	0.15
	Kwashiorkor	8(31)	4(14)	0.19
	Desquamation	2(8)	1(3)	0.60
	HIV positive*	9(35)	14(48)	0.65
**Severity**	**N (%)**			
	Deep breathing	18(69)	21(72)	0.88
	Hypoxia (<95% or unrecordable)	3(12)	3(10)	0.57
	Tachypnoea (>60 brpm)	16(62)	13(45)	0.22
	Severe tachycardia (>160 bpm)	11(42)	8(28)	0.25
	Capillary refill ≥3 s	15(58)	16(55)	0.53
	Weak pulse volume	13(50)	19(66)	0.24
	Bradycardia (<60 bpm)	0	0	
	Temperature gradient	17(65)	23(79)	0.25
	Hypotension (SBP <70 mmHg)	5(19)	5(17)	0.44
	WHO shock criteria	18(69)	23(79)	0.39
	Hypothermia (ax. temp < 35°C)	0	0	
**Hydration**				
	Reduced skin turgor	8(31)	16(55)	0.07
	Sunken eyes	11(42)	19(66)	0.08
**Consciousness**				
	Coma	3(12)	4(14)	1.00
	Prostration	15(58)	13(45)	0.34
**Abnormal biochemistry N (%)**				
	Acidosis (base deficit >8)	12(46)	20(69)	0.09
	Creatinine (>80 μmols/L)	13(50)	13(49)	0.70
	Hypokalaemia(<3.0 mmols/L)	16(62)	19(66)	0.76
	Hyperkalaemia(>5.5 mmols/L)	2(7)	0	0.22
	Hyponatraemia(<125 mmols/L)	4(15)	3(10)	0.58
	Hypernatraemia (>145 mmols/L)	3(12)	3(10)	1.00
	Hypoglycaemia(<3.0 mmols/L)	1(4)	4(14)	0.36
	Hyperglycaemia(>10.0 mmols/L)	0	0	
**Mean laboratory variable, ± SD**				
	Haemoglobin, g/dl	8.7(2.2)	8.9(1.9)	0.67
	pH	7.25(0.25)	7.26(0.13)	0.79
	Base deficit, mmol/L	14(11)	17(6)	0.26
	Creatinine, μmol/L	107(78)	95(58)	0.53
	Bicarbonate, mmol/L	14(13)	10(5)	0.16

*7 children were missing HIV test results: 4(15%) HSD/5D; 3(10%) RL arms

### Primary outcomes

#### Resolution of shock

**Figure 2:**
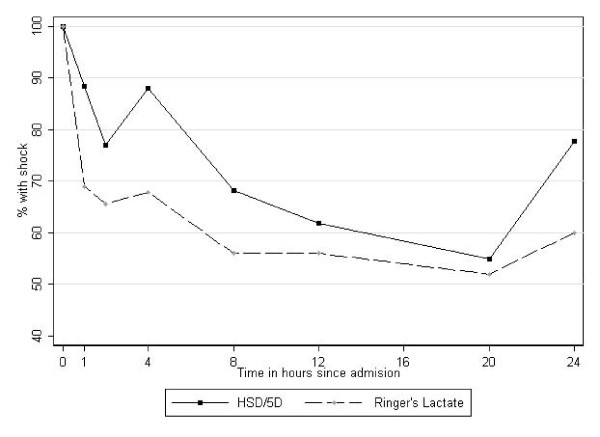
**Proportion of children in shock over 24 hours of observation**: A higher proportion of children randomised to HSD/5D (WHO solution) remained in shock compared those receiving RL over 24 hours of observation.

By 8 and 24 hours the proportion of children with shock in both RL arm and HSD/5D was considerable, 14/25(56%) and 15/22(68%) respectively. A larger decline in the proportion with shock was observed in RL recipients compared to HSD/5D particularly in the diarrhoeal group however these differences were not significant at any time point (Table 2 and Figure 2).

**Table 2:** Primary and Secondary outcomes

	Time	HSD/5D (n = 26)	RL (n = 29)	p
**PRIMARY OUTCOMES**				
Number with shock, n/N(%)	8 h	15/22(68)	14/25(56)	0.39
	24 h	14/18(78)	14/25(56)	0.14
Oliguria (<1 ml/kg/hour), n/N(%)	8 h	9/22(41)	3/25(12)	0.02
	24 h	8/18(44)	6/25(24)	0.16
Tachycardia (>160 bpm), n/N(%)	8 h	6/22(27)	4/25(16)	0.34
	24 h	8/14(44)	4/25(16)	0.04
Creatinine, mean(± standard deviation)	8 h	112(85)	104(60)	0.73
	24 h	89(56)	112(87)	0.39
**SECONDARY OUTCOMES**				
Tachypnoea (>60 br.pm), n/N(%)	8 h	7/22(32)	2/25(8)	0.04
	24 h	7/18(39)	3/25(12)	0.04
Base deficit, mean(± standard deviation)	8 h	10(13)	15(7)	0.16
	24 h	12(8)	8(9)	0.38
In-hospital mortality, n/N (%)		15/26(58)	13/29(45)	0.34

#### Tachycardia

Persistent tachycardia (an index of unresolved shock) was common at 8 and 24 hours, was present in 10/47(21%) and 12/39(31%) children respectively, but was more prevalent in the HSD/5D arm (p = 0.04) (Table 2). Median AUC of the heart rates were similar for both study interventions (Kruskal-Wallis: χ^2 ^= 0.3; p = 0.59).

#### Oliguria

**Figure 3:**
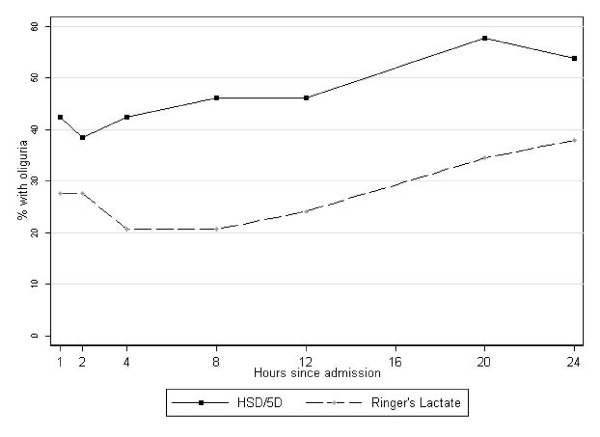
Proportion of children with oliguria over 24 hours of observation

Since renal failure was excluded as entry criteria we used adequate urinary output as a gold standard for successful fluid resuscitation; with oliguria being a marker of persistent, severe shock. Oliguria was common in both intervention arms but more prevalent in the HSD/5D arm at 8 hours (9/22; 41%) than in those receiving RL (3/25; 12%) p = 0.05. By 24 hours, oliguria was still common children receiving HSD/5D regime but no different from RL group (Table 2 and Figure 3). Median AUC for the hourly urine output (ml/kg/hr) was lower in HSD/5D recipients (51; IQR, 36, 116) than RL recipients (101; IQR, 63, 141) (Kruskal-Wallis χ^2 ^= 4.6; p = 0.03). Mean serum creatinine levels and proportion with elevated creatinine were similar in both arms over the course of admission.

### Secondary outcomes

#### Respiratory distress

Severe tachypnoea was less common in the children receiving RL than HSD/5D at both 8 and 24 hour. Mean respiratory rate was greater in the HSD/5D arm than RL arm at 8 and 24 hours (p = 0.002) (Table 2). Overall, there was a trend towards higher median AUC of respiratory rates was noted in those who died (2262; IQR, 1938, 2897) compared to survivors (2015; IQR, 1547, 2391) (Kruskal-Wallis: χ^2 ^= 3.6; p = 0.06).

#### Resolution of base deficit

The base deficit at 8 hours in children receiving HSD/5D was lower than the RL (p = 0.04) but no difference was noted in survivors to 24 hours (p = 0.81). Many trial participants remained severely acidotic (base deficit >15 mmol/L) over the first 24 hours (Table 2). Comparing the admission and 24 hours base deficit (% change over baseline) we noted that the mean reduction was greater in those who received RL (8.5 mmol/L; p = 0.002) as compared HSD/5D (1.0 mmol/L; p < 0.87).

#### Mortality

Overall, 31/61(51%) children died; HSD/5D (15/26 = 58%); RL (13/29 = 45%) and HAS (3/6 = 50%) (p = 0.62). The difference in mortality between those receiving HSD/5D and RL was non-significant (p = 0.34). Of the children who died, 84% (26/31) fulfilled the WHO malnutrition shock definition at admission. Case fatality rate in this high risk subgroup was 59% (26/44), irrespective of allocated intervention, and was associated with an increased risk of death (Risk Ratio = 2.0, 95% confidence interval 0.92-4.36; P = 0.05) compared to those that did not have these criteria. In the group with severe diarrhoea mortality was higher 13/19(68%) in those receiving HSD/5D than in those receiving RL 9/22(43%) p = 0.11. Case fatality in the group with presumptive septic shock was 2/7(43%) for HSD/5D was and 4/8(50%) for RL (p = 0.61). Thirteen (42%) of the fatalities were HIV positive, 14(45%) were HIV negative, and 4 (13%) declined HIV tests. Infection with HIV did not significantly increase the risk of death (odds ratio 1.18; 95% confidence interval 0.38, 3.72; p = 0.76). Nine (29%) of the deaths were in children who had kwashiorkor, case fatality was 69% (9/13) in children with kwashiorkor, irrespective of intervention arm. Kwashiorkor was associated with a non significant increased risk of death (odds ratio 2.2; 95% confidence interval 0.7, 10.1; p = 0.14).

#### Time to death

Thirty nine percent (12/31) of the deaths occurred within 24 hours of recruitment while 52% (16/31) of fatalities occurred within 48 hours of enrolment. On Kaplan Meier survival analysis, we found no significant difference in the time to death when any of the intervention fluids are used for resuscitation (logrank test: combined (p = 0.42).

#### Severe adverse events

No child developed clinical features of pulmonary oedema or allergic reaction (to HAS) during the course of study observation. Frusemide or other diuretics were not required or prescribed during the trial. There were no differences in the mean sodium concentration at admission (133[SD ± 11] versus 134[10]; p = 0.81), 8 hours (134[10] versus 139[10]; p = 0.09), and 24 hours (138[9] versus 140[9]; p = 0.47) between those who received HSD/5D and RL respectively.

## Discussion

Our observations of the WHO severe malnutrition shock management protocol using half-strength Darrow's in 5% dextrose (HSD/5D) in the pilot study and Phase II trial indicate a very high mortality when applied rigorously and with some improvement when applied to a lower risk shock group and with changes to supportive management strategies (100% and 58% respectively). Shock was inadequately corrected evidenced by persisting shock in 78% and oliguria in 40% of survivors to 24 hours during the RCT. Cautious fluid resuscitation using low dose isotonic solution (RL) was shown to be safe, with moderately better resolution of some, but not all, of the haemodynamic parameters of shock, but without significant survival advantages. By 24 hours 50% of children in the RL arm had persisting feature of shock, including 24% with oliguria. Cardiogenic failure, evidence by pulmonary oedema, was not observed for any of the fluid resuscitation strategies in this trial.

The high overall mortality, 51% and inadequate correction of shock in all study arms, resulted in a decision to prematurely terminate the trial, and followed consultation with the external safety monitors. Owing to the safety concerns over the use of fluid resuscitation in children with severe malnutrition the compromise design of the trial including low to modest volume fluid expansion and a protocol with little flexibility over additional boluses. Nor did the design purposely comply with either the WHO guideline for non-malnourished children or international accepted paediatric practice. As the trial progressed the absence of any clear evidence of cardiogenic failure and volume overload coupled with the high mortality raised anxieties over the rationale for withholding standard international paediatric practice guidelines. Moreover, there was reluctance by the clinical team to continue enrolment into a trial with a compromised intervention strategy and a concern that adoption of the standard approach to fluid resuscitation was increasingly becoming the more justifiable approach.

The significant departure of the WHO malnutrition shock treatment recommendations from accepted paediatric practice without adequate physiological or clinical evidence is a major concern. Early and aggressive resuscitation of children with isotonic fluids has shown substantial survival benefits. For example, it has been demonstrated that for every hour that shock is left uncorrected leads to a doubling of mortality [16,17]. This latter observation has relevance to our experience and results of our FIM trial. The pilot and the FIM trial included severely malnourished children diagnosed and managed in accordance with current WHO guideline in whom there was a universally fatal outcome indicating that intervening at this stage is 'almost too late'. With the amendment to entry criteria, where shock was defined using a less stringent definition, we found a lower but still unacceptably high mortality.

The decision to undertake this trial and its early termination reflect the difficulties encountered when strongly promoted international guidelines might be considered at odds with current best clinical practice, albeit largely derived from experience in quite different geographical and cultural settings. We demonstrated that even in children with kwashiorkor, the outcomes were not worse in those receiving isotonic fluids. Our findings are consistent with findings of a recent study from Bangladesh where children with severe malnutrition and cholera safely tolerated up to 100 ml/kg of isotonic fluid (cholera saline) given 6 hours[18]. Although cholera represents a special case, with disproportionately huge fluid loses, the findings of both these studies challenge the notion that children with severe malnutrition have myocardial dysfunction together with sodium (and water) retention[19] rendering them susceptible to incipient cardiogenic failure and inability to cope with rapid volume expansion using isotonic fluids[8]. Studies that have reported reduced mortality following adoption of the fluid management guidelines, and hence used to justify the current fluid recommendations, have involved concurrent introduction a whole care package and therefore have many confounders since they were not designed to answer the specific question of fluid resuscitation[4,20-23]. Even these studies have studies have reported inconsistent findings.

The origins of these concerns date back to studies from 1960^s ^and 1970^s ^indicating that children with severe malnutrition have a state of 'reductive adaptation' with sodium and water retention, expanded extracellular compartment, myocardial atrophy and a 'hypocirculatory state' said to recover on nutritional rehabilitation if intravenous fluids are avoided [2,24-29]. Supportive evidence was drawn from radiographic studies showing reduced cardiothoracic ratios on x-rays, autopsy studies showing diminished heart size, and histological changes such as interstitial oedema and myocardial atrophy[30,31]. Whereas other observers concluded that the heart is reduced in size in concordance with the skeletal musculature[28,29] and both systolic and diastolic functions are well preserved, indicating that response to fluid expansion should be similar to non-malnourished children[32,33]. The observation of the expanded extracellular space (ECF) was challenged by Fronius suggesting that the apparent expansion of ECF was spurious and resulted from the relative changes in the intracellular compartment, which disproportionately contracts in severe wasting[34]. What is clear from the literature is that very few studies linked clinical status, physiological investigation and response to treatment in representative cohorts of children with severe malnutrition. Few have drawn on modern technology and contemporary understanding of paediatric critical illness to study myocardial status and haemodynamic response to fluid expansion. Our reading of the literature and guidelines suggests that there is substantial confusion with respect to the understanding of the complex interaction of myocardial dysfunction and circulatory failure (hypovolaemia), well recognised in paediatric critical illness, with that of heart failure and circulatory overload when the terminology is frequently used synonymously[26]. Much of the work examining total body sodium concentration and impaired kidney function were usually conducted in inadequately clinically described study cohorts and focused on single organ pathophysiology [35-38] unlinked to haemodynamic status or whole body physiology. This may lead to different interpretation of the findings, for example, in the early stages of shock or dehydration sodium and water retention are a common compensatory mechanism in severe illness with circulatory impairment[39].

In the absence of physiological studies it is difficult to challenge these viewpoints, which underpin the scientific rationale advanced for the current WHO fluid resuscitation guidelines. We conclude that since the assumed risks of heart failure did not materialise in our trial we suggest that this treatment priority may have been given disproportionate emphasis and is based on insufficient scientific evidence to justify withholding standard fluid resuscitation practice.

Our study, together with recent trial in malnourished Bangladeshi children, provide new findings to add to the scientific literature and extend the debate with regards to fluid resuscitation in children with severe malnutrition[8,40-44]. The high mortality, persistence of shock and oliguria suggests that more aggressive treatment may be warranted. Whilst research is undertaken to define the most appropriate guideline, the most pragmatic approach for clinicians when faced with this very challenging clinical scenario is probably to follow the standard of care outlined by the WHO for non-malnourished children [6,7,11,45,46]. However, caution will still be required while introducing the use of higher volumes of isotonic fluids than presently recommended by WHO malnutrition guidelines[8]. Our study also did not include a systematic assessment myocardial dysfunction and therefore relied on clinical diagnosis of fluid overload. Newer techniques for monitoring haemodynamic response to fluids, which are easy to learn, non-invasive, reliable and reproducible are now available and could be easily used in low resource settings[47]. These techniques offer assessment of myocardial function at presentation together with monitoring of haemodynamic response to fluid expansion. Finally, our findings do not exclude further consideration of colloids in children complicated by septic shock, since the HAS arm was too small to draw any meaningful conclusions.

## Conclusion

Despite many shortcomings, this is the first randomised controlled trial comparing isotonic fluids to one of the hypotonic fluids recommended by WHO for treatment of shock in children with severe malnutrition. Volume expansion using isotonic fluids were at least as safe as hypotonic solutions recommended in the current guideline since cardiogenic failure did not complicate their use. Shock was not adequately corrected by any of the fluid strategies and poor outcome lead us to conclude that the volumes and rate of correction of hypovolaemia currently recommended and used in this trial are inadequate. Although use of isotonic fluid was associated with modest improvement in shock and survival when compared to HSD/5D, these results remain inconclusive. Future research should consider standard WHO or international fluid resuscitation recommendations in these children.

## Competing interests

The authors declare that they have no competing interests.

## Authors' contributions

**SOA **helped design and execution of the study, data analysis, interpretation of trial data, wrote the initial draft, and was involved in finalising the manuscript. **JK and MB **helped design and execution of the study; and contributed to the interpretation of the trial data and writing of the manuscript. **PN **was the study statistician involved in the analysis and interpretation of data. **KM **conceived and designed the study. She was involved in the data analysis, interpretation and manuscript drafting and revising it critically for important intellectual content and wrote the final draft. All authors read and approved the final manuscript.

## Pre-publication history

The pre-publication history for this paper can be accessed here:

http://www.biomedcentral.com/1471-2431/10/71/prepub
